# Identifying the Needs for a Web-Based Postpartum Platform Among Parents of Newborns and Health Care Professionals: Qualitative Focus Group Study

**DOI:** 10.2196/16202

**Published:** 2020-05-26

**Authors:** Lyzette T Laureij, Leonieke J Breunis, Regine P M Steegers-Theunissen, Ageeth N Rosman

**Affiliations:** 1 Department of Obstetrics and Gynecology Sophia Children's Hospital Erasmus University Medical Center Rotterdam Netherlands; 2 Department of Health Care Studies Rotterdam University of Applied Sciences Rotterdam Netherlands

**Keywords:** newborn, focus groups, postpartum period, postnatal care, eHealth, pregnancy, obstetrics, qualitative research

## Abstract

**Background:**

During the turbulent postpartum period, there is an urgent need by parents for support and information regarding the care for their infant. In the Netherlands, professional support is provided during the first 8 days postpartum and for a maximum of 8 hours a day. This care is delivered by maternity care assistants (MCAs). Despite the availability of this extensive care, a majority of women prefer to make use of a lesser amount of postpartum care. After this period, access to care is less obvious. Where parents are automatically offered care in the first 8 days after birth, they must request care in the period thereafter. To compensate for a possible gap in information transfer, electronic health (eHealth) can be a valuable, easily accessible addition to regular care.

**Objective:**

We explored the needs and preferred content by new parents and health care professionals of a web-based platform dedicated to the postpartum period and identified barriers and facilitators for using such a platform.

**Methods:**

We conducted 3 semistructured focus groups among (1) parents of newborns, (2) MCAs, and (3) clinicians and administrators in maternity care. A topic list based on a framework designed for innovation processes was used. Thematic content analysis was applied.

**Results:**

In the focus group for parents, 5 mothers and 1 male partner participated. A total of 6 MCAs participated in the second focus group. A total of 5 clinicians and 2 administrators—a member of a stakeholder party and a manager of a maternity care organization—participated in the third focus group. All user groups underlined that a platform focusing on the postpartum period was missing in current care, especially by parents experiencing a gap following the intensive care ending after the first week of childbirth. Parents indicated that they would perceive a postpartum platform as a proper source of reliable information on topics regarding breastfeeding, growth, and developmental milestones, but also as a tool to support them in seeking care with appropriate professionals. They also emphasized the need to receive personalized information and the opportunity to ask questions via the platform. MCAs acknowledged added value of providing additional information on topics that they address during the early postpartum period. MCAs as well as clinicians and administrators would guide parents to such a platform for additional support. All user groups experienced disadvantages of using an authentication procedure and filling out extra questionnaires to receive tailored information.

**Conclusions:**

Our research shows that parents of newborns, MCAs, and clinicians and administrators foresee the additional value of a web-based postpartum platform for at least the whole postpartum period. The platform should be easily accessible and personalized. Content on the platform should contain information regarding breastfeeding, growth, and developmental milestones. A chat function with professionals could be considered as an option.

## Introduction

Pregnancy, delivery, and taking care of the infant during the postpartum period are large life events. In many countries, the postpartum period (ie, the first 42 days after childbirth) is a largely underestimated period of care for parents [1,2]. It is known that they are in enormous need for information, especially regarding breastfeeding, sleeping patterns, and physical recovery after childbirth [3]. To support maternal and infant health and to prevent morbidity and mortality at the earliest moment during the life course, it is essential that this information is available and accessible [3-5].

In the Netherlands, postpartum care in the first days after birth is provided by trained professionals, so-called maternity care assistants (MCAs), supervised by community midwives (see Textbox 1 for more information). An MCA provides care for an average of 8 consecutive days, 3-8 hours a day, at the home of the parents of the newborn. All women are offered this extensive postpartum care. However, the majority of women choose to receive fewer hours or days of care than recommended [6-8].

After these first 8 days, care is less extensive and less regularly scheduled at well-baby clinics for the newborn, which are free of charge and are organized by preventive child health care (PCHC) services. The mother has only one regular postpartum checkup with the midwife at 6 weeks postpartum. This less-extensive care requires women to actively ask for support and guidance. Electronic health (eHealth), for example, a web-based platform, has the potential to support continuity of care for both parents and professionals. It is already widely used in supporting disease management, promoting healthy lifestyles, prevention, and making care more effective [9,10]. There are more eHealth tools focusing on pregnancy than in any other medical field [11,12]. Nevertheless, there is a lack of any eHealth program focusing on the postpartum period. This is a missed opportunity, because it is known that women are willing to use eHealth applications for reliable information during this new phase in life [13,14].

In countries other than the Netherlands, postpartum care is less extensive. However, independently taking care of an infant at home after early discharge from a maternity ward, without the help of a professional, is experienced as very difficult and stressful [15-17]. Parents of newborns also need compassion and companionship, as loneliness and psychological problems are main issues in the postpartum period [18]. In addition, there is a need for continuity of professional care during transition of the prenatal to postpartum period [18-22]. eHealth, in its broadest sense, can be a useful additional technique to provide tools for support in this period for parents [13,23-25]. Additionally, health care professionals recognize problems with handover of information in this period, leading to suboptimal care [26].

**Maternity care in the Netherlands.**

**Care During Pregnancy**

In the Netherlands, maternity care is a complex care system. Pregnant women are allocated to three strata of care: primary, secondary, or tertiary care. Allocation is based on division of low-, medium-, or high-risk pregnancy during the first prenatal visit. Primary care is provided by the community midwife to women who have a low-risk pregnancy. Women may give birth at home or at a primary care birth center, supervised by the community midwife.

Secondary care during pregnancy and childbirth takes place at general hospitals by gynecologists or clinical midwives. Women with high-risk pregnancies (eg, severe preeclampsia prior to 32 weeks of gestation) are referred to tertiary care, provided by 12 perinatal centers in the Netherlands.

During the third trimester, maternity care assistants (MCAs) visit every woman at home for an assessment of the recommended amount of postpartum care. Postpartum care is provided by MCAs working at maternity care organizations. These are independent enterprises.

**Care During the Postpartum Period**

Regardless of the strata of care, women are offered postpartum care by MCAs. This care is supervised by the community midwife. Most women choose to receive postpartum care at home, but this is also possible at primary care birth centers. If there are maternal or neonatal complications, part of this postpartum care can take place at the maternity ward (eg, 48 hours of stay at the maternity ward is recommended after a cesarean section). Care by MCAs is provided during the first 8-10 succeeding days. This care includes coping with the new situation and increasing parents’ confidence in the care for the infant [6]. They also promote a healthy lifestyle, such as preventing use or reuse of tobacco, and educate parents in the prevention of child abuse [4]. Most of the information and advice is transmitted orally, and information leaflets are used for further support [6].

The mother herself decides the amount of postpartum care, up to a maximum recommended by the MCA. The minimum amount is 24 hours and the recommended amount in a standard situation is 49 hours (eg, when a mother breastfeeds her baby and no problems occur during childbirth or the early postpartum period). During the first few days, postpartum care covers 6-8 hours a day. During the following days, this number of hours is reduced. On the last day, the care for the newborn is transferred to well-baby clinics of preventive child health care (PCHC) services. The woman remains under the supervision of the community midwife until 6 weeks postpartum.

Care by midwives, both community and clinical and during both pregnancy and the postpartum period; gynecologists; and PCHC services is covered by a compulsory health insurance. Postpartum care by the MCAs is partly covered by health insurance and women are required to pay an out-of-pocket amount of €4.40 ($4.77 US) per hour, as of 2019, of receipt of this form of postpartum care.

It is unclear whether women who already receive extensive postpartum care would also appreciate a web-based postpartum platform and what the content should be. Also, health care professionals, especially in postpartum care, are not familiar with using eHealth to support their work. Therefore, the aim of this study was to explore the needs and preferred content by new parents and health care professionals of a web-based platform dedicated to the postpartum period and to identify barriers and facilitators for using such a platform.

## Methods

### Overview

Textbox 2 details the concept of the proposed web-based postpartum platform to support continuity of care for both parents and health care professionals.

**Concept of the web-based postpartum platform.**

**Background**

Women tend to have a need for easily accessible information and support during the preconception period and pregnancy. Electronic health (eHealth) has proven its potential to support women and their partners, such as in achieving a healthier lifestyle and gaining information about pregnancy and healthy lifestyle [10,25]. A web-based platform focused on the postpartum period could be an additional support for women and their partners during the postpartum period. When this web-based platform is also available for obstetric professionals and maternity care assistants (MCAs), women who have a low postpartum care uptake may be reached more easily. A web-based platform has some advantages above other forms of eHealth, such as lower development costs, less instability caused by upgrades, and easier access for all involved health care professionals.

**Aim of the Web-Based Platform**

The web-based platform must be an addition to regular postpartum care by MCAs for parents of the newborn. The platform focuses on information provision and prevention.

**Target Group**

The platform should target parents of newborns and health care professionals, including maternity care professionals, MCAs, and preventive child health care (PCHC) service professionals. Parents will look for reliable information on the web-based platform and professionals will provide this reliable information.

### Design and Setting

The design of this study was a qualitative approach with focus group interviews. The focus groups were conducted in February 2018 at the Erasmus University Medical Center (Erasmus MC) – Sophia Children’s Hospital, a university medical center in Rotterdam, the Netherlands.

### Study Participants

#### Overview

Two types of users of this web-based postpartum platform were identified: (1) parents of newborns and (2) health care professionals. Parents with a child younger than 12 months were asked to participate as a target group of future users of the platform (ie, parents of newborns), hereafter referred to as parents. Several health care professionals are involved in the care during pregnancy, childbirth, and the postpartum period. We decided to split the health care professionals into two groups: MCAs and all other professionals, hereafter referred to as clinicians.

MCAs were approached to participate as potential ambassadors but also as future users of the platform during their working routine. MCAs fulfil a different role in the care for new parents than the other maternity care professionals. Their role is of a more caring nature, as they are specialized nurses. Midwives, obstetricians, and PCHC physicians have less-intensive contact with parents of newborns and are more at a distance. Additionally, MCAs work under supervision of community midwives and may feel that they cannot express themselves freely in the presence of the other maternity care professionals.

Finally, clinicians such as gynecologists, midwives, and PCHC physicians were invited to participate because of their potential contribution to the content of the platform. Also, administrators involved in perinatal care, such as managers of maternity care organizations and the Dutch Patient Federation, were invited because of their potential role in the dissemination of the platform. This group will be referred to as clinicians and administrators.

To increase objectivity and prevent barriers in expressing subjective opinions and experiences, we arranged separate focus groups. All participants were recruited by email and telephone via existing networks from other studies in the Erasmus MC - Sophia Children’s Hospital, such as the Rotterdam periconception cohort (Predict Study) and the Healthy Pregnancy 4 All-2 (HP4All-2) study [27,28]. We aimed to recruit 6-10 participants per focus group. In advance of the meeting, all participants received information regarding participation in a focus group, web links to existing pregnancy-related platforms, and statements on the subject.

#### Parents

Inclusion criteria for parents were (1) having a child born in the past 12 months before the focus group meeting took place, (2) being 18 years of age or older, and (3) having a sufficient understanding of the Dutch language. After women gave consent to participate, a member of the research team called them to ask whether their partner also wanted to join the focus group.

#### Maternity Care Assistants

MCAs were recruited via managers of maternity care organizations. They met inclusion criteria if they (1) had a sufficient understanding of the Dutch language and (2) worked as an MCA.

#### Clinicians and Administrators

Clinicians were eligible to participate if they worked in PCHC services or obstetric care. Also, managers of maternity care organizations and the Dutch Patient Federation were asked to participate.

### Data Collection

Before the start of the focus groups, each participant was asked to fill out a questionnaire on baseline characteristics. An experienced and trained researcher (ANR) guided all three focus groups and two research assistants took notes. Each focus group was audiotaped and started with a short introduction explaining the aim of the study. Participants were reassured of confidentiality and encouraged to speak freely.

The focus groups were semistructured and based on a topic list grounded in a framework developed by Fleuren et al [29]. This theoretical framework was developed to explain determinants of innovation within health care and recognizes four main stages of innovation processes: dissemination, adoption, implementation, and continuation. In this study, we especially focused on the first two stages: dissemination and adoption. Transition from one stage to the next can be affected positively or negatively by four different factors: the end user, the innovation itself, the sociopolitical environment, and characteristics of the organization. In order to obtain a complete overview of potential barriers and facilitators, these factors were the main themes of the topic list. Subthemes were based on literature research [12,23,30-32] and divided among the corresponding main themes.

The first part of the focus groups entailed a discussion about the needs for a postpartum platform and wishes for the content of the platform (ie, general information, specific topics, and sources). Secondly, the look and feel of the platform was discussed by showing different existing platforms regarding pregnancy or older children (see Multimedia Appendix 1 for the topic list).

### Data Analysis

All transcripts were transcribed verbatim and were anonymized. The verbatim transcriptions were checked with the original audiotapes for accuracy by LTL and returned for member checking to the participants. We used the qualitative software program NVivo 11 (QSR International) to support data analysis. We intended to analyze the results according to the theoretical framework based on the model of Fleuren et al [29]. However, this framework appeared to be inefficient due to too much overlap in coding. Thematic analysis was then applied. The three different transcripts were independently coded by two researchers (LTL and LJB) to create a set of preliminary codes. These preliminary codes were discussed with a third researcher (ANR) and consensus was reached on the codes per transcript. Thereafter, these codes were arranged into different main themes and subthemes for each of the transcripts. The themes, subthemes, and codes were compared between the three groups to check for similarities and differences. Themes and subthemes corresponded between the different groups, although there were differences in preferences. Therefore, the codes in the main themes and subthemes were divided into facilitators and barriers by LTL and LJB. Finally, consensus was reached on the subdivision by the three researchers (LTL, LJB, and ANR).

### Ethical Considerations

The Medical Ethics Committee of Erasmus MC declared that the rules laid down in the Medical Research Involving Human Subjects Act, also known as its Dutch abbreviation WMO, do not apply to the study protocol (NL/s/MEC-2017-1134). All parents provided written informed consent; the need for written informed consent from the MCAs as well as clinicians and administrators was waived.

## Results

### Participants

A total of 5 mothers and 1 male partner joined the focus group for parents (see Table 1). All mothers received postpartum care for at least 24 hours.

**Table 1:** Characteristics of participants: parents of newborns.

Sex	Age (years)	Marital status	Education level	Ethnicity	Parity	Age of youngest child (months)	Complicated pregnancy or childbirth	Place of most recent delivery	Amount of postpartum care
Female	32	Married	High	Caucasian	1	7	No	General hospital	Recommended amount
Female	27	Married	High	Caucasian	1	3	Yes	General hospital	Less than recommended amount
Male	26	Married	High	Caucasian	N/A^a^	7	N/A	N/A	N/A
Female	34	Together, but living apart	Moderate	Caucasian	2	9	No	General hospital	Less than recommended amount
Female	33	Married	High	Caucasian	1	8	No	General hospital	Recommended amount
Female	31	Cohabiting	High	Non-Caucasian	1	9	Yes	General hospital	Recommended amount

^a^N/A: not applicable.

In the MCA focus group, 6 MCAs participated (see Table 2). They worked at different maternity care organizations and their work experience varied from 3 to 26 years. The third focus group (ie, clinicians and administrators) consisted of 5 clinicians working in perinatal care, 1 manager of a maternity care organization, and 1 employee of the patient federation (see Table 3).

**Table 2:** Characteristics of participants: maternity care assistants (MCAs).

Sex	Age (years)	Ethnicity	Work experience (years)
Female	48	Caucasian	26
Female	54	Caucasian	8
Female	35	Caucasian	3
Female	53	Non-Caucasian	14
Female	50	Caucasian	3
Female	31	Caucasian	6

**Table 3:** Characteristics of participants: clinicians and administrators.

Sex	Age (years)	Ethnicity	Profession	Work experience (years)
Female	24	Caucasian	Manager at maternity care organization	6
Female	38	Caucasian	Community midwife	15
Female	46	Caucasian	Gynecologist	6
Female	40	Caucasian	Clinical midwife	17
Female	65	Caucasian	Preventive child health care physician	40
Female	32	Caucasian	Policy officer at Dutch Patient Federation	3
Female	37	Caucasian	Community midwife	15

### Emerging Themes

We identified three different main themes regarding the need and content of a postpartum period platform: (1) information on platform, (2) additional facilities, and (3) accessibility. These three main themes were divided into various subthemes, and facilitators and barriers within these subthemes were identified (see Table 4). The main themes and various subthemes are described in detail in the Results section.

**Table 4:** Main themes, subthemes, facilitators, and barriers.

Main themes and subthemes		Facilitators	Barriers
**Information on platform**			
	General	Need for a postpartum platform^a,b,c^ Providing general information^a,b,c^ Categorized by period^a,b,c^ Uniformity among information given by health care providers^a,b^ Statistics on outcomes^a^	For general population^a,b,c^ Superficial information^a,b^ Registration or extra questions^a,b^
	Care guidance	Advice on when to contact professional^a,b,c^ Care guidance for domestic abuse^b^	N/A^d^
	Information topics	Psychosocial support, sleeping, crying, breastfeeding and bottle feeding, food, birth control and fertility postpartum, and older children^a,b,c^ Healthy food and diet-specific information^a,b,c^ Prevention^a,b,c^	No moderator^a^ Recipes^a,b^
	Sources	Reliable sources^a,b,c^ Visibility of the sources^a,b^	Funded by industry^c^
**Additional facilities**			
	Communication	Chat function and consultation of professional^a,c^	Fixed amount of push messages^a,b^
	Findability of information on the platform	Frequently asked questions on general topics^a^ Search option^a,c^	N/A
**Accessibility**			
	Language use	N/A	Complicated language^a,b,c^ Mandatory tone^c^
	Look and feel	Images and footage^b,c^	Too much text^c^ Formal layout^c^ Shabby layout^a,b,c^
	User group	Vulnerable population^b,c,e^	N/A
	Findability and guidance	Easily findable^a^ Guidance to platform by obstetric professionals^a,b,c^	Unreliable forums are easy to find^a^ Maternity care assistants (MCAs) not trained in electronic health (eHealth)^c^
	Timing	Preconception to 6 months postpartum^a,b,c^ Filling gap between MCA and preventive child health care (PCHC) service^a,b^	Starting during pregnancy or postpartum period^a^
	Authentication	Access without authentication^a,c^ Anonymous^a,b,c^	Obligation to create an account and authentication^a,b,c^
	Costs	N/A	Paywall^a,b,c^
	Device	App^a,b,c^	Email^a,b,c^

^a^Facilitators and barriers applicable to parents.

^b^Facilitators and barriers applicable to maternity care assistants (MCAs).

^c^Facilitators and barriers applicable to clinicians and administrators.

^d^N/A: not applicable.

^e^Vulnerable populations include underserved populations with multifactorial problems, including health illiteracy.

### Information on Platform

#### General

All user groups indicated that a platform dedicated to the postpartum period should be an all-around platform with a wide range of information, dedicated to and classified by period of pregnancy, childbirth, and the postpartum period. All parents ensured that there is a need for general information brought to them passively (eg, not in periodic emails). MCAs stated that the platform would be a good support for parents during the postpartum period. The involvement of different professionals in the whole postpartum period, such as the community midwife, MCA, general practitioner, and PCHC service, leads to a feeling of discontinuity of care among parents, according to all user groups. Parents added to this statement that they often heard different advice from different maternity professionals and PCHC services.

Both parents and MCAs acknowledged the added value of a platform that provided the same information as MCAs, offering parents a possibility to reread orally given information.

Parents also expressed the need for a platform that collected all existing reliable information of other websites. Clinicians and administrators agreed on this. All user groups stressed the existence of several platforms about pregnancy, focused on information for a general population, and that they missed personalized information; for example, specific information on how to address certain problems with the infant (eg, sleeping problems) or psychological problems as a new mother. Filling out extra questions in order to receive personalized information was, however, perceived as bothersome. This was supported by MCAs. In contrast, all groups indicated that filling out extra questions on diet and receiving personalized advice to improve their diet would be desirable. Parents and MCAs feared that generalized information could lead to anxiety among parents if their infant did not reach a developmental milestone. A solution for this was brought up by parents: providing statistics on the incidence of certain preconditions or milestones could be helpful.

Err what I... for example, the statistics say 10 percent of [postpartum] women gets a postpartum depression. Well, if you hear that then you think, “All right, it’s not that unlikely that I don’t feel well occasionally.”

Parent focus group, female

#### Care Guidance

All groups experienced barriers among parents to contact the appropriate professional when problems occurred. Clear information on the platform on when to contact which professional was suggested. One of the aspects MCAs are responsible for is sharing specified information on topics regarding domestic abuse and violence. MCAs often share their private telephone numbers—although officially not allowed—with women suspected of being a victim of domestic violence. MCAs thought that providing lists of institutions and telephone numbers in different languages on the platform might be helpful and safer for themselves.

Also, MCAs felt that sometimes they had to provide information on prevention (eg, shaken baby syndrome and postpartum depression) too early, and if they could refer to the platform, new parents could read the information again and use the information when needed.

And it also applies to me personally, that if I would search for something [on the web-based platform] and the advice on the platform would be “consult a health care professional,” then I would be more encouraged to eventually call [the professional].

Parent focus group, male

#### Information Topics

Parents stated that a platform dedicated to the postpartum period should contain specific information on several main topics regarding the mother and the infant. They felt that information on psychosocial support, physical recovery, and birth control was important. Regarding the infant, sleeping patterns, crying, and breastfeeding or artificial milk were key topics.

Clinicians and administrators mainly recognized the physical recovery as an important topic, while MCAs named breastfeeding. All user groups also suggested that the platform should contain specific information on healthy food for both mother and infant. Extra information on diet-specific information (eg, vegetarian lifestyle) was desired. Providing healthy recipes was perceived as a barrier by parents and MCAs, because this was found to be culture specific. Parents suggested that they would prefer specific information on healthy food for their infant, such as which vegetables should be introduced first. All user groups stated that coaching by periodic emails containing messages, questions, tips, and facts on a healthy lifestyle and maintaining a healthy lifestyle may be less well-placed during the postpartum period.

Both clinicians and administrators as well as parents emphasized the added value of the possibility to share experiences with other new parents. Parents commented that posting about experiences online often leads to negative comments by other parents. They thought it would scare people off if the experiences and replies were not moderated by a professional.

#### Sources

The information on a platform must be reliable and must be composed by decent sources, such as professionals from a hospital or professional organizations. These sources should be visible on the platform to increase the sense of reliability. This was stated by clinicians and administrators, as well as by MCAs. Parents confirmed this, and also emphasized that commercial sources reduced the perceived reliability of the platform.

No, those websites where you can see “this text is revised by a certain lung specialist so-and-so, from such-and-such hospital.” Then I acknowledge that written text more than Parents From Now [a commercial magazine with a website, focused on young parents: Ouders van Nu, in Dutch], which is sponsored by Zwitsal [Dutch baby products brand]...

Parent focus group, female

### Additional Facilities

#### Communication

Provision of information via the platform was also discussed. MCAs and parents mentioned the possibility of push messages. Parents felt that push messages regarding healthy lifestyle were too demanding and too paternalistic. They suggested the possibility to adjust the frequency of messages. Clinicians and administrators added that push messages following a parent’s question on the platform would be more convenient, and MCAs stated that messages should have a positive tone.

Both parents as well as clinicians and administrators advised to provide a chat function with a professional on the platform that would be focused on acute problems. This could be used to reassure parents regarding topics such as crying, but also to guide parents to the right professional. The clinicians and administrators remarked that the responder needed to be a trained professional. They suggested 24-hour coverage, as they experienced parents calling them during the night with problems, while parents preferred a chat function during the daytime until early in the evening.

#### Findability of Information on the Platform

MCAs suggested reserving a part of the platform for frequently asked questions (FAQ). Parents proposed that the questions asked most frequently during a chat conversation could be collected and added to a FAQ topic on the platform.

In order to increase the findability of information on the platform, the platform should have a search option. This was experienced as essential by parents as well as clinicians and administrators, so that the platform becomes more personalized.

Because at the moment you have a chat function [on the web-based portal], you will notice which questions are asked more. And then a commonly asked subject, for example, I would really like to have that [collected] in a question-and-answer database. So, accordingly, one can search for questions or complaints.

Parent focus group, male

### Accessibility

#### User Group and Language Use

All user groups agreed on the use of accessible language. Clinicians and administrators perceived the use of patronizing language as a potential barrier for parents. All user groups underlined that the use of scientific words scared off potential users. MCAs as well as clinicians and administrators stated that a platform was particularly desirable for a vulnerable population with low health literacy. They agreed that the language had to be adjusted to that population accordingly, even though clinicians and administrators doubted whether this vulnerable population could be reached by a platform, as illustrated in the following quotes.

I think [use of] language is also very important.

Respondent #1, clinician and administrator focus group

Yes, just basic language.

Respondent #2

Yes, that you will not be patronized.

Respondent #1

Yes, [language level] B1, everything as much as possible in B1, yes. And explain difficult words by, for example, clicking on it. A lot of images.

Respondent #3

#### Look and Feel of the Platform

The look and feel of the platform was found to be very important by all user groups. This influenced the degree of attractiveness for the users. Clinicians and administrators as well as parents underlined the importance of a neat layout. Shabby look and feel as well as a too-formal layout were considered to be barriers for parents to visit the platform. Clinicians and administrators, especially, emphasized the use of images and footage instead of large amounts of text, in order to reach parents with lower health literacy skills.

#### Findability and Guidance to the Platform

In order to reach different populations, parents underlined that the platform must be easily found on online search engines. They said that unreliable forums pop up more often in search engines, and this might result in fewer people finding the platform.

Parents also indicated that if their obstetric professionals would advise them to visit the platform, that would increase the findability and value of the platform. They missed the guidance to additional information such as eHealth during regular care, especially when they were transferred multiple times between the strata in maternity care.

Clinicians and administrators as well as MCAs also stated that guiding parents to an approved platform would be better than letting parents find information on the internet themselves. Also, MCAs perceived that some of their colleagues were not able to work with a platform.

If my doctor would have said or something that I should do it [visiting the web-based platform], then I think I would have... if you get the advice to do it, then I would make the effort to do so.

Parent focus group, female

#### Timing

Parents indicated that they were more likely to change their lifestyle prior to pregnancy. They stated that they would more often use the platform for obtaining information during their pregnancy and postpartum period if they already used it before pregnancy. They also felt that the platform should provide information until 6 months after childbirth.

MCAs as well as clinicians and administrators saw the regular checkups during pregnancy as an important moment to refer to such a platform. MCAs underlined that parents need more guidance, particularly in the gap between the first week postpartum after the MCA has left and the start of more intensive guidance by the PCHC service.

And I notice that the gap, so to say, from the end of the postpartum care by MCAs until [the start of] the PCHC services, those four weeks, that is actually too long.

Respondent #1, MCA focus group

Yes.

Several other respondents

#### Authentication

A perceived barrier among all user groups regarding the accessibility of the platform was the obligation to create an account and log on (ie, authentication). The possibility to ask questions anonymously on the platform was preferred by all user groups. MCAs as well as clinicians and administrators experienced problems with other platforms when they had to create an account and thought that would be a problem for parents also.

The moment I have to log on and create an account with a password, it puts me off.

Parent focus group, female

#### Costs

The same barrier was perceived regarding paying for using the platform. Parents said that a free platform would be preferred, but if it was really useful, they would consider paying a small amount of money to gain access. Both clinicians and administrators as well as MCAs feared a paywall; they thought that, in particular, the population they wanted to reach with the platform—the vulnerable population—would not be reached if they had to pay.

Look, I work with very different families [during the first week postpartum], I work with families that, so to say, can’t even buy a half bread, and with well-off families. Yes, you know, the communication lines [with health care professionals] are shorter, especially compared to those who have money problems.

Respondent #1, MCA focus group

Yes, and especially for those people—

Respondent #2

—you need... you need this [web-based postpartum platform].

Respondent #1

You really need this.

Respondent #3

#### Device

Finally, it was discussed in all three focus groups that the platform should be mobile-phone friendly. Parents said that during breastfeeding they often check their mobile phones and that this would be a great moment to search for information. MCAs as well as clinicians and administrators pointed out that even among the poor families, almost everybody has a mobile phone with internet access and that sending messages to their phones would be more convenient than emailing.

## Discussion

### Principal Findings

In order to develop an eHealth platform to be used by new parents but also by maternity care professionals, we aimed to explore the need for and content of a web-based platform to be used during the postpartum period. Our research showed that there is a need for such a platform, preferably until 6 months after childbirth in addition to regular postpartum care. The platform and the information on the platform should be easy to find. Also, platform developers should pay special attention to the look and feel of a platform in order to increase the usability. Topics on the platform should focus on general information about pregnancy, childbirth, and the postpartum period, but also on more personalized information. A difficulty with this is that parents emphasized the need for personalized information, but they also have a problem with authentication and filling in additional questions about their personal situation; therefore, personalization of information was limited.

### Strengths and Limitations

One of the strengths of this study was the safe environment created by arranging three separate focus groups guided by an experienced moderator. Additionally, all participants were given the opportunity to express their opinions and experiences equally. Another strength was the proper qualitative health method that was used for the focus groups and analysis of the data. Furthermore, by using a framework approach, a clear topic list was used to guide the discussions in which all facets of innovation were covered. The transcripts were independently coded by two researchers, resulting in a high level of intercoder agreement.

In addition, all potential user groups of a postpartum period platform were represented. By including not only parents, MCAs, and midwives but also PCHC professionals and administrators, we had the opportunity to consider the need for a postpartum platform and the content from all perspectives. This contributed strongly to the usability and robustness of our results.

In terms of limitations, there is a possible selection bias. The participants in the parent focus group were generally of Caucasian origin and highly educated. Despite intensive attempts, only one partner, who was male, participated. This may influence the external validity of the results. On the other hand, the MCAs added rich descriptions of their experiences with clients with low socioeconomic status that were in line with the opinions expressed by the parents. Therefore, the overall influence of selection bias on the results may be limited. Another limitation of this study is that some topics were only briefly discussed due to time limitations and, therefore, depth is lacking on some topics. However, by using this approach we were able to cover a wide range of topics. This enabled us to investigate the preconditions for such a platform from a broad perspective.

### Comparison With Prior Work

All user groups stated that there is a need for a platform dedicated to the postpartum period because continuity of care is missed and parents hear different advice from different professionals. Problems with handover of information and care among professionals in maternity care has gained more awareness, but was not discussed in our focus groups [26]. The feeling of a lack of continuity of care and receiving conflicting advice among parents is also supported by Baas et al [33].

Furthermore, it is well known that women experience stress, loneliness, insecurity, and feelings of isolation after childbirth [1,31]. eHealth could provide a partial solution to this problem [13,18,23,34]. However, parents in our focus group felt that eHealth is more important for access to fast and reliable information than to solve feelings of loneliness. A possible explanation can be the presence of the MCA during the postpartum period. Additionally, parents would like to use a platform dedicated to the postpartum period up to several months after childbirth.

The needs for the content of the platform were in line with the findings of Slomian et al [23]. General topics, such as information regarding breastfeeding, physical recovery after childbirth, postpartum depression, among others, were mentioned [23]. Accordingly, the underlying need for reassurance and empowerment was also mentioned and this is recognized among other postpartum women around the world [2]. All user groups acknowledged the added value of care guidance to the appropriate professional and it is known that eHealth can contribute to this process [9]. Push messages were experienced as essential in order to receive important information but also as irritating by parents if the content does not match topics that are important to them and could lead to extra stress. This equilibrium has been recognized in previous research [35,36]. In addition to Slomian et al, we showed that even women who receive extensive postpartum care prefer the use of a platform during the postpartum period [23,34].

Parents find it stressful if they cannot contact a professional directly [36]. A chat function is required and this may reduce the stress. This chat function does not require 24-hour coverage according to the parents, in contrast to the findings of Danbjørg [36]. The intensive presence of an MCA during the first week postpartum in the Netherlands may be an explanation for this difference.

In general, the knowledge on the importance of a healthy lifestyle before conception and during pregnancy for both mother and infant is increasing [37-39]. eHealth has the potential to support women to achieve a healthy lifestyle during the preconception period and pregnancy [12,40]. However, parents in our focus group study rejected the idea of achieving a healthy lifestyle with the use of eHealth specifically during the postpartum period and would rather see a platform with information about factors other than lifestyle, such as physical recovery and sleeping patterns of an infant. A combination of both might strengthen the platform.

To ensure the usage of the platform, maternity care professionals should guide women and their partners toward this platform, especially vulnerable women and their partners. Commercial companies already use online websites to inform pregnant and postpartum women about their products and are very experienced with this concept. Collaboration with these commercial companies may increase the knowledge on proper ways of attracting parents to the platform.

Future research should focus on cost-effectiveness and improvement of quality of care of such a platform, since it is an addition to regular postpartum care. Also, the needs and accessibility of a postpartum platform for vulnerable parents or parents with low health literacy should be taken into account in further research. For example, separate interviews with these parents could be undertaken, especially to adapt the content of the platform to their needs and preferences. A randomized controlled trial could be undertaken, targeted to a vulnerable population, in order to investigate the relationship between reliable information via a platform and maternal empowerment.

### Conclusions

Parents and involved maternity care professionals foresee a need for a web-based postpartum period platform, despite the presence of MCAs during the first week after childbirth. This web-based platform ideally connects to preconception and pregnancy platforms and is accessible until 6 months after childbirth, and parents should be referred to this platform by professionals working in perinatal care. There is a need for information provision that is both easily accessible and reliable. Information on the platform should focus on general topics, such as breastfeeding, psychological support, and physical recovery after childbirth. However, the web-based platform should also be tailored to the individual needs of the parents and a chat function is advised. eHealth in the form of a web-based platform may be a suitable solution to this.

## Appendix

Multimedia Appendix 1 Topic list for a postpartum platform.

## Abbreviations

eHealth: electronic health
Erasmus MC: Erasmus University Medical Center
FAQ: frequently asked questions
HP4All-2: Healthy Pregnancy 4 All-2
MCA: maternity care assistant
PCHC: preventive child health care
WMO: Medical Research Involving Human Subjects Act
