# Supplementary material for: Identifying the Needs for a Web-Based Postpartum Platform Among Parents of Newborns and Health Care Professionals: Qualitative Focus Group Study
Source: JMIR Form Res. 2020 May 26;4(5):e16202. doi: 10.2196/16202 (PMC7284398; doi:10.2196/16202)
Supplement: Multimedia Appendix 1 [file formative_v4i5e16202_app1.docx]

**Multimedia Appendix 1
Additional Table 1.** Topic list

| Factors that may influence stages of innovation processes | Main theme | Subtheme | Extra information |
| --- | --- | --- | --- |
| The End User | Information (active and passive) | Nutrition Exercise Psyche General topics on pregnancy, childbirth and postpartum | Mother Child |
|  | Look and Feel |  |  |
|  | Target group | Language  Linguistic   Partner Culture Diet | Dutch, English, multiple languages Health literacy Imagery Language level  Vegan/Vegetarian Culture based diet |
|  | Sources | Chat function References |  |
|  | User period | Start Duration |  |
|  | Current offer |  |  |
|  | Accessibility | Log on Questionnaires |  |
|  | Commitment |  |  |
| The Innovation | Information (active or passive) | Nutrition Exercise Psyche General topics on pregnancy, childbirth and postpartum |  |
|  | Look and Feel |  |  |
|  | Sources | Chat function References Reliability |  |
|  | User period | Start Duration |  |
|  | Accessibility | Findability Device Notifications All round platform | All information on one platform |
|  | Degree of use |  |  |
|  | Relevance of information |  |  |
|  | Possibilities of interconception care |  |  |
| The Socio-political environment | Privacy | Legal Log on Questionnaires |  |
|  | Costs | Small compensation |  |
|  | Health insurer | Preventive care |  |
| The Organisation | Guiding professionals to the platform |  |  |
|  | Guiding platform to professionals |  |  |
|  | Professionals | Postpartum care |  |
|  | Care pathways |  |  |

Topics used for structuring the focus groups based on the stages of innovative processes by the model of Fleuren [29].
